# Efficacy of bisphosphonates in detection of early enamel caries using NIR fluorescence imaging and inhibition of caries progression

**DOI:** 10.7150/ijms.60013

**Published:** 2021-06-11

**Authors:** Jie Sun, Mindy Gil, Shahrzad Khorashadi, George Chen, Cliff Lee, Yoshiki Ishida, Masazumi Nagai, Shinichiro Wada, Shigemi Ishikawa-Nagai, John D Da Silva

**Affiliations:** 1 Restorative Dentistry and Biomaterial Sciences, Harvard School of Dental Medicine, USA.; 2 Oral Medicine, Infection and Immunity, Harvard School of Dental Medicine, USA.

**Keywords:** caries detection, NIR fluorescence imaging, bisphosphonates, caries inhibition

## Abstract

NIR fluorescence imaging using bisphosphonate-Indocyanine green has been indicated for early interproximal caries detection. This study assessed diagnostic accuracy of caries detection by NIR fluorescence imaging with OsteoSense 750^®^ (OS750) *in vitro* and *ex vivo*, and to analyze the therapeutic efficacy of a bisphosphonate (Etidronate) in inhibiting enamel caries progression *in vitro*.

**Methods**: Four experiments were conducted using extracted human teeth; 1) to calculate the infiltration rate of OS750 into interproximal white spot lesions using fluorescence microscope, 2) to assess diagnostic accuracy of interproximal natural white spot lesions using desktop NIR fluorescence imaging device *in vitro* setting, 3) to assess diagnostic accuracy of artificially created deeper enamel carious lesion (0.5 mm~1.0 mm) using NIR fluorescence image through the head-mount display in *ex vivo* setting, 4) to compare the progression on the enamel caries lesions treated by Etidronate, NaF and distilled-water. Diagnostic accuracy was analyzed using sensitivity, specificity and receiver operating curves (ROC). The caries progression was calculated with micro-CT and was statistically analyzed using a two-way ANOVA and the Tukey HDS post-hoc test.

**Results**: 1) The infiltration rate of OS750 was 101.83% ± 8.66 (Min: 90.10%, Max: 133.94%). 2) The average of sensitivity and specificity *in vitro* setting experiments were 86.7% ± 4.4% and 70% ± 11%, respectively. The average of area under the ROC curves (AUC) was 0.883 ± 0.059 indicating excellent performance. 3) The mean sensitivity and specificity in *ex vivo* setting was 82.97% ± 15% and 76.78% ± 13.27% respectively. 4) The carious lesion volume treated by Etidronate was significantly smaller at post treatment-1 (p<0.05) and treatment-2 (p<0.01) than the control. There was no significant difference in lesion volume in the Etidronate and NaF group at the time point of post treatment-1.

**Conclusion**: This study suggests that bisphosphonates contribute to both early diagnosis of enamel caries and inhibition of caries progression.

## Introduction

Although the prevalence of dental caries has been declining worldwide in the last few decades, it remains a serious chronic disease and public health issue 1, 2. Nearly 25% of adults from age 20 to 64 in the United States currently live with untreated dental caries 3. Early caries prevention is critical and treatment is the most effective at an early stage. A safe, non-radiographic method sensitive enough to consistently and reliably detect early-stage carious lesions would enable early diagnosis 4, 5, 6, 7, 8, 5 and patient-tailored preventive therapy 6.

The new paradigm of operative conservatism, sometimes referred to as “minimally invasive dentistry,” incorporates the dental science of detecting, diagnosing, intercepting and treating dental caries at the microscopic level 7. There is a critical unmet need for a non-invasive optical caries detection system that will enable both early detection and simultaneous treatment of early interproximal caries. Current methods for caries detection using visual-tactile examination and radiographs detect only advanced lesions with a depth of at least 500 µm in the enamel, leaving incipient caries undetected 8. Furthermore, interproximal caries can only be detected by radiograph, which usually significantly underestimates the actual size or depth of a carious lesion 9. By the time a lesion becomes visible on a bitewing X-ray, the only effective therapy is invasive class II restoration, potentially subjecting the tooth to a lifetime of treatment. Moreover, the conventional bitewing X-ray procedure exposes the patient to ionizing radiation. In fact, the collective doses to patients arising from the medical use of ionizing radiation continue to rise significantly year to year; it has been shown that the cumulative risk of cancer at age 75 from diagnostic X-rays is directly linked to the annual exposure frequency 10.

Our previously published study indicated the potential of NIR fluorescence imaging using bisphosphonate-Indocyanine green (ICG) agent for detection of the early enamel interproximal caries. The NIR fluorescence imaging agent OsteoSense 750^®^ (OS750) showed excellent properties in infiltration rate and intensity representative of the magnitude of the carious lesion. The sensitivity and specificity for interproximal were 0.86 and 0.8 using OS750 and 0.34 and 0.9 using bitewings 11.

Bisphosphonates, an ICG-Fluorescence imaging agent, are a class of organic compounds with a variety of pharmacologic actions, most notably used in the treatment of osteoporosis 12-14. It has been reported that bisphosphonate derivatives, such as MHDP (methane-hydroxy-diphosphonate) and EHDP (ethane-1-hydroxyl-1, 1-diphosphonate), are effective in inhibiting dissolution of enamel 14-15. Previous studies have shown that bisphosphonates are able to bind with high affinity to exposed hydroxyapatite in enamel and can inhibit hydroxyapatite breakdown 16-18. Specifically, Fleisch & Russel found that bisphosphonates reduce the incidence of carious in rats and mouthwashes containing bisphosphonates reduce calculus deposition in humans 17. Gandolfi *et al* found that alendronate solution pH 5.0 and 7.4 both inhibited enamel dissolution significantly from acid attacks 16. Studies focusing on the effect of bisphosphonates on hydroxyapatite structure have shown that bisphosphonates are able to alter the hydroxyapatite crystal morphology in a dose-dependent manner 19-20. We believe that this high binding affinity of bisphosphonates could provide two benefits: inhibit further demineralization of enamel carious lesions and promote remineralization at deeper sites that fluoride is unable to penetrate.

Etidronate is a first-generation bisphosphonate characterized by its short alkyl chain and absence of nitrogen that has a high affinity for demineralized hydroxyapatite 10, 21. The chemical formula of Etidronate is similar to the bisphosphonate portion of OS750^®^ which is an NIR bisphosphonate fluorescence imaging agent. NIR fluorescence imaging has been used to capture osteoblastic activity 22-24. It is used in the imaging of the lymphatic system for diagnosis and assessment of function which is accomplished with NIR using indocyanine green 25. Our previous study indicated 93% infiltration depth rate of OS750^®^ into deep enamel caries lesions, and has desirable chemical and optical properties with a high potential as an NIR imaging agent in early caries detection 11.

The purpose of this study is to further assess the optical properties and diagnostic accuracy of caries detection using NIR fluorescence imaging with OS750 *in vitro* and *ex vivo*, and to analyze the therapeutic efficacy of a bisphosphonate (Etidronate) in inhibiting enamel caries progression *in vitro*.

## Materials and Methods

**Figure 1:**
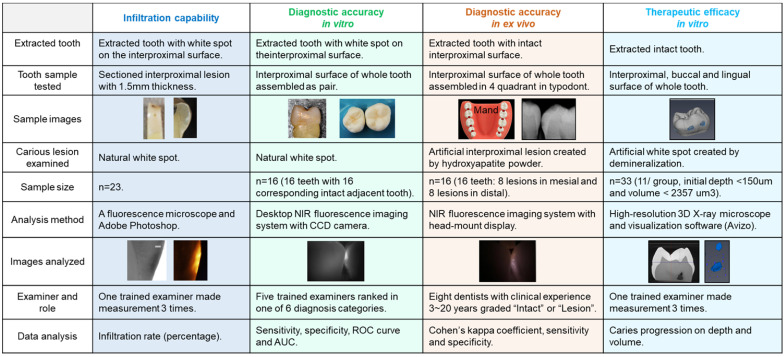
Experimental scheme of assessment of Infiltration capability, diagnostic accuracy *in vitro*, diagnostic accuracy in *ex vivo* and therapeutic efficacy *in vitro*.

**Figure 2:**
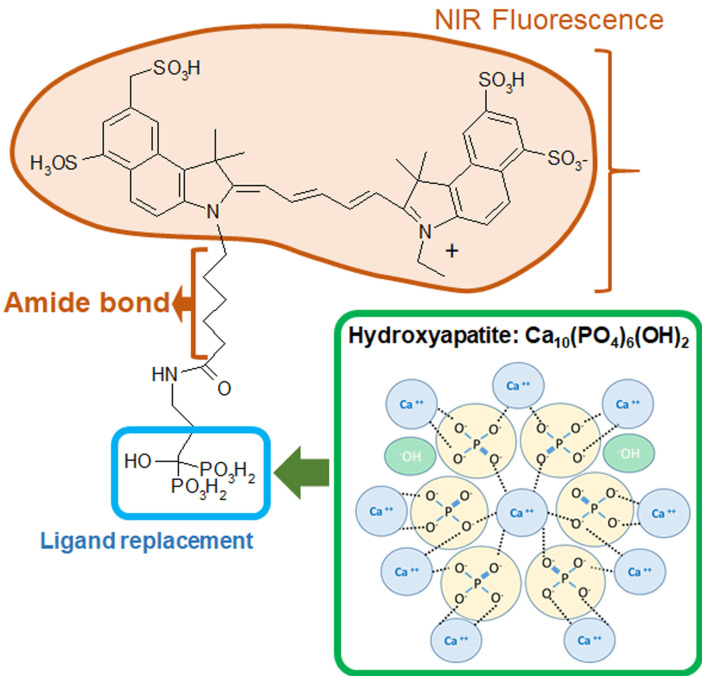
Formula of OsteoSense750 and binding mechanism with hydroxyapatite.

Four experiments were conducted using extracted human teeth (Figure 1). OS750 (PerkinElmer, Waltham, MA, USA) which is an NIR bisphosphonate fluorescence imaging agent used to detect early enamel carious lesions (Figure 2). Etidronate (Etidronate disodium hydrate, Sigma-Aldrich, USA: Group ET) was used for the therapeutic purpose to inhibit carious progression.

This study was approved by the Institutional Review Board (IRB) of Harvard Medical School. Extracted human premolars and molars without any cavitated interproximal lesions were collected from the Harvard Dental Center. The roots of each tooth were removed and the coronal portions were physically cleaned of any debris or soft tissue using periodontal scalers.

### Infiltration capability of OsteoSense 750^®^ into natural interproximal white spot lesions

**Figure 3:**
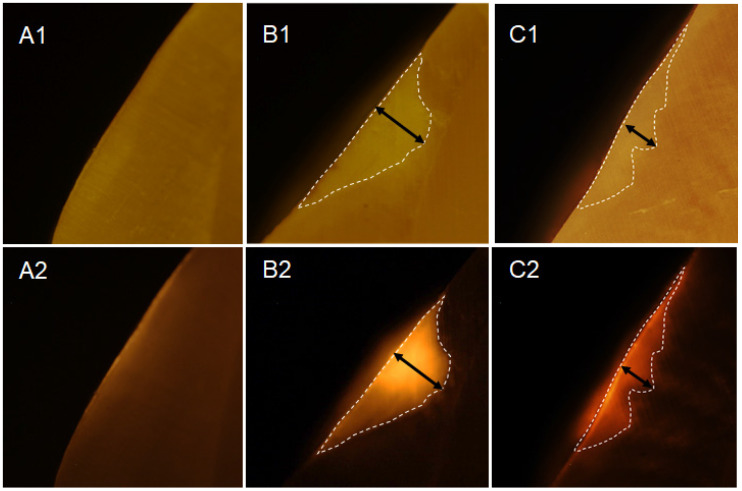
** Infiltration of OS750 into the lesion.** A1 and A2 represent microscope image of the intact tooth surface with regular (A1) and fluorescence mode (A2). B1, B2 and C2, C2 indicate the enamel lesion.

Twenty-three white spot lesions observed on the interproximal surface of the extracted human teeth were used for this analysis (Figure 1). Each ROI (region of interest) was treated by 3 µl of OS750 (OS750, 1:100 dilution) for 30 seconds, then rinsed with doubled distilled water (ddH_2_O) for 10 seconds and allowed to dry. Each tooth was abraded from the buccal-lingual surface to a 1.5 mm section that included the ROI using Buehler Vector Powerhead (Isomet 1000: Buehler, USA), then further polished to approximately 1.0 mm section. A fluorescence microscope BX51 (Olympus America, Philadelphia, USA) was used to capture two different images of each specimen: a histological image under a light microscope setting of 4X power (Figure 3-A1, -B1, and -C1) and a fluorescent image with a 750 nm excitation filter and 770nm emission filter (Figure 3-A2, -B2, and -C2). The area of the carious lesion in the light microscopic image and fluorescence signal on the fluorescence image were measured using Adobe Photoshop CS5 measurement logs. The average of 3 measurements made by the same examiner was used for data analysis. The ratio of OS750 infiltrated into the carious lesions were calculated (Ratio = area of fluorescence signal on the fluorescence-microscope image/area of carious lesion on the light-microscope image × 100) and Spearman's correlation coefficient between OS750 infiltration ratio and the area of carious lesion was analyzed. Sample size (p = 0.05, power = 0.90) was calculated 11.

### Diagnostic accuracy of interproximal caries using NIR imaging with OS750 in an *in vitro* setting

**Figure 4:**
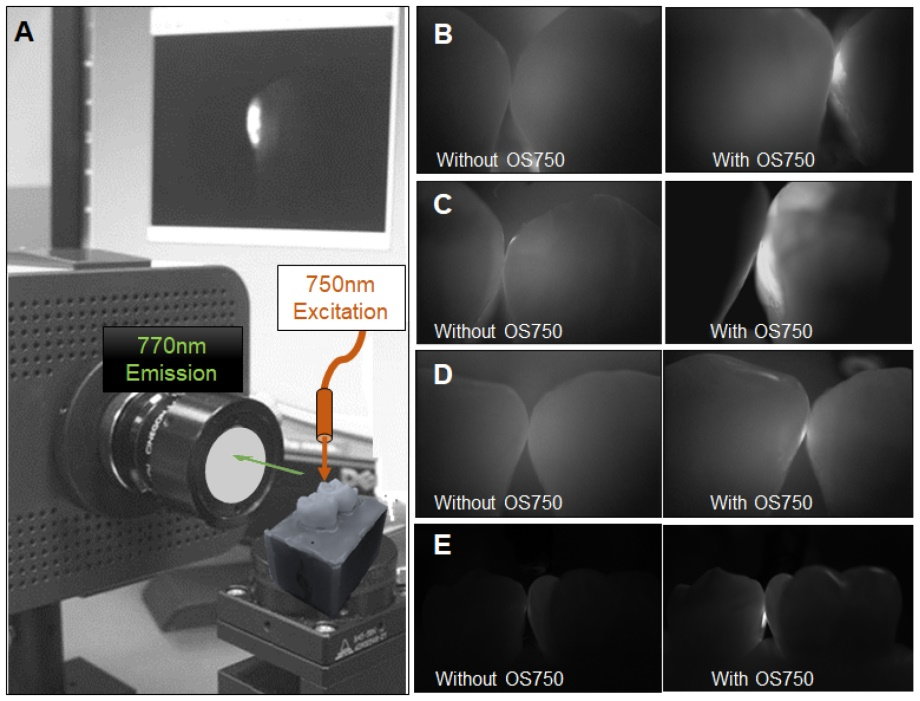
** Fluorescence imaging system and observed images with OS750. A:** The system is comprised of CCD camera, sample stage, excitation light and computer display. The tooth with ROI was on the stage with corresponding adjacent tooth placed. The excitation light was illuminated on the marginal ridge of ROI and fluorescence images was captured by CCD camera with 770 nm emission filter from the buccal side perpendicularly. **B-E:** Captured images without and with OS750.

Sixteen human extracted teeth (8 premolars and 8 molars) with interproximal natural white spot lesions and intact buccal and lingual surfaces were used (Figure 1). Each region of interest was treated with 3 µl of OS750 (1:100 dilution) in the same manner. The whole tooth and corresponding adjacent intact tooth were paired and stabilized with interproximal contact using silicon impression material (EXAMIX, GC America, USA). Each of the 16 pairs were placed on the stage of the fluorescence imaging system (Figure 4A) with the buccal side facing the Charge-Coupled Device (CCD) camera (MC 285SPD-L0B0: Texas Instrument, USA). The 750nm, Xenon light source (MAX301 Asahi Spector, Japan) to illuminate the marginal ridge of the interproximal area (Figure 4A). Real-time images of the emission fluorescence signal coming through the buccal surface were captured by the CCD camera (Figure 4B-E). Five trained evaluators assessed images and ranked in one of 6 categories (Definitely Positive, Probably Positive, Possibly Positive, Negative, Probably Negative, and Definitely Negative). Receiver operating curves (ROC) of each evaluator was made and the area under the curve (AUC) was compared. For the gold standard, fluorescence signal existence was defined as positive. Sample size (p = 0.05, power = 0.90) was calculated 11.

### Diagnostic accuracy of interproximal caries using NIR imaging with OS750 in an *ex vivo* setting

**Figure 5:**
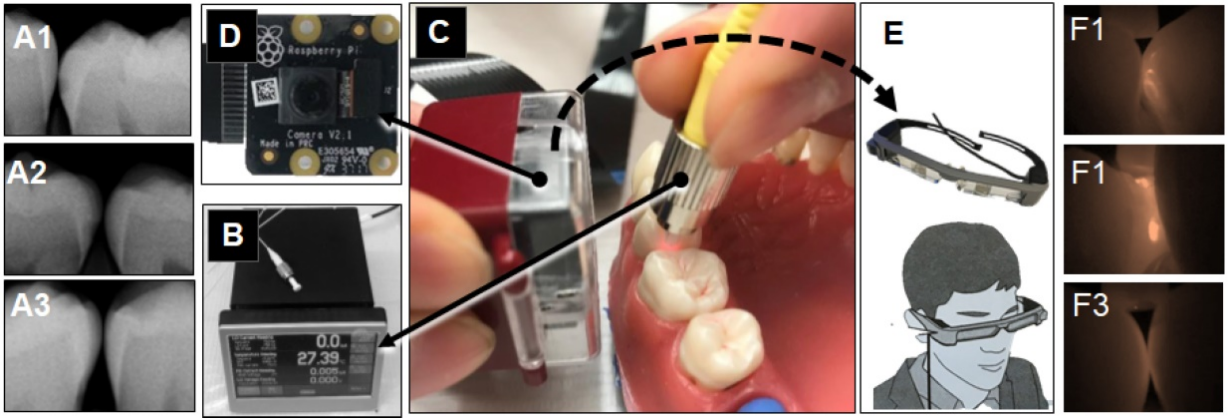
** Caries imaging system using the head-mount display. A:** radiograph (A1: intact, A2 and A3: mock lesion), **B and C:** The Near-Infrared (NIR) illumination light (750nm, Laser diode) was excited over the interproximal area at 45~90 degree angle. D: Emission fluorescence was captured by the CCD camera (780nm) from the buccal side. **E:** The signal was sent to the head-mount display. **F1 and F2** shows NIR images for the interproximal lesion,** F3** indicates image of intact surface.

Extracted intact human teeth (n=20) were assembled in the typodont from the second molar to the canine for each of 4 quadrants and a total of 32 interproximal surfaces were used for the assessment (Figure 1). A small lesion with approximately 1.0 mm diameter and 0.5~1.0 mm depth was created on 16 interproximal surfaces using a #2 carbide bur. These holes were packed with the hydroxyapatite powder (mock lesion, Figure 5-A1, -A2). Another 16 interproximal surfaces were kept as intact (Figure 5-A3). Two microliters of 1/100 dilution of the stock solution of OS750 was applied to all the interproximal surface lesion. The NIR illumination light (730nm, Laser diode, Fig. 5B) was excited over the interproximal area at 90-degree angle (Figure 5C). Emission fluorescence was captured by the CCD camera (780nm, Figure 5C and 5D) and visible on a head-mount display (Figure 5E, 5-F1, 5- F2, and 5-F3). Two dentists repeated the assessment 3 times with 1 week in between and repeatability was calculated as Cohen's κ. Eight dentists with clinical experience of 3~20 years examined each interproximal surface and graded them as either “Intact” or “Lesion” using NIR images and the sensitivity, specificity and inter-examiner error were calculated. Sample size (p = 0.05, power = 0.90) was calculated 11.

### Therapeutic efficacy of etidronate on the inhibition of enamel caries progression

**Figure 6:**
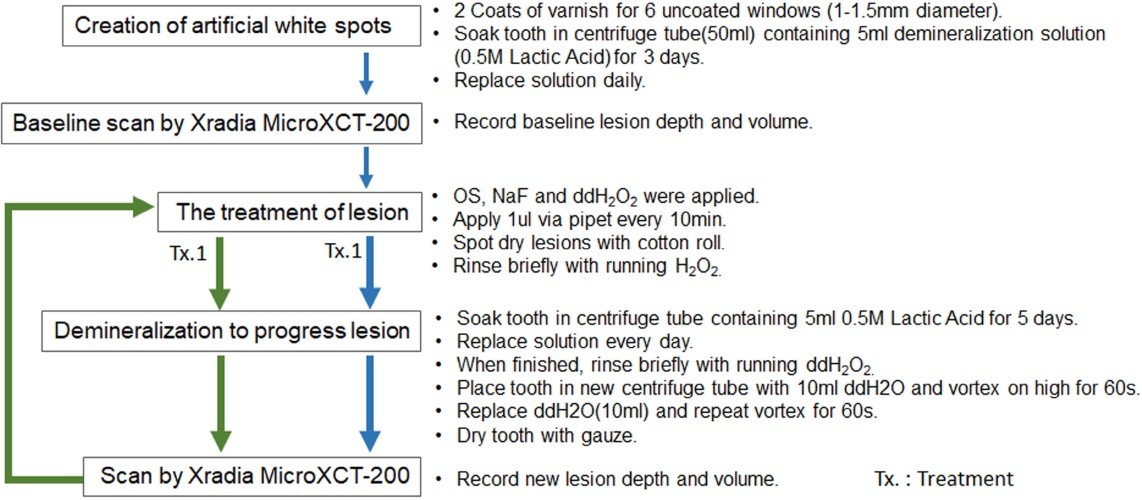
Experiment protocol.

**Figure 7:**
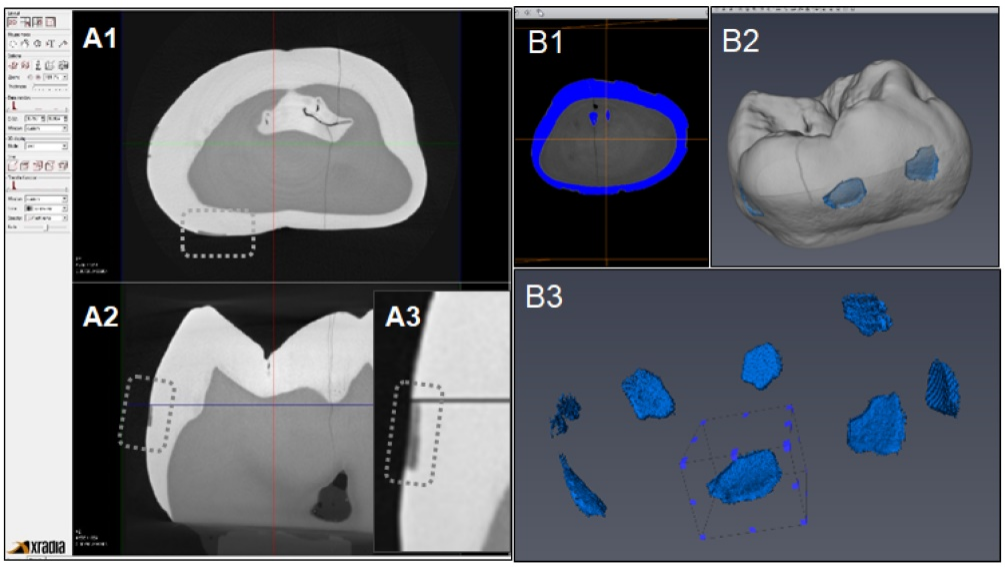
** CT images acquisition and measurement of lesion volume using Aviso. A1:** Xradia images reconstructed. **A2:** Shallow lesion were observed in the axial surfaces. **A3:** Enlarged image of lesion. **B1:** Original image for Aviso. **B2:** The 3D presentation of lesions. **B3:** The volume on each lesion were calculated.

Figure 6 indicates the experimental protocol. Fifty-six artificial lesions were created on intact flat surfaces (interproximal, buccal and lingual) using 12 extracted human teeth (Figure 1). Two coats of enamel varnish were applied to the flat surface of each tooth, leaving 4 ~ 6 exposed windows of approximately 2.0 mm diameter. Each tooth was then submerged in a 50ml centrifuge tube containing 5 ml of 0.5 M lactic acid demineralization solution for 3 days and the solution was replaced every 24 hours. Each tooth was rinsed with doubled distilled water (ddH_2_O: DD) twice for 60 seconds each time. The lesion depth was measured using a high-resolution 3D X-ray microscope (Xradia MicroXCT-200, Zeiss, Figure 7 -A1, -A2, and -A3). All teeth were scanned using the same filter, magnification, exposure time, kV and voltage. Following reconstruction, lesion depths were calculated using Avizo 8.1 (Figure 7 -B1, -B2 and -B3). A total of 33 lesions with initial depths of 150 µm (±21.3) and initial volume of 235 E+07 µm^3^ (46.5) were used for the experiment.

The lesions were randomly divided into 3 treatment groups: Fluoride (Oral B Minute-Foam: Group NaF), Etidronate (Etidronate disodium hydrate, Sigma-Aldrich, USA: Group ET) or Control (Group: DD). For the group NaF, 2 µL of NaF foam was applied to each lesion every 10 minutes for 3 times. For the group ET, Etidronate disodium hydrate in 10% preparation in ddH_2_O applied with same manner as the fluoride group. After the treatment, each tooth was gently rinsed with ddH_2_O and immersed in the demineralization solution (0.5 M lactic acid) for 5 days, with the solution being replaced daily. At the 5-day mark, each tooth was placed in a new centrifuge tube with 10ml ddH_2_O and vortexed on high for 60 seconds twice, replacing the ddH_2_O in between each run. A CT scan of each tooth was then obtained using the Xradia MicroXCT-200. Two rounds of treatment, demineralization, and CT scans were performed. Following the image reconstruction of each tooth, the average lesion volume at baseline, post treatment-1 and post treatment-2 were calculated using Avizo 8.1. The lesion volume on each of 3 groups at baseline, post treatment-1 and post treatment-2 by 2 treatment materials were compared by Two-way ANOVA and the Tukey HDS post-hoc test. Samples size was determined with reference tested alendronate treatment 16.

## Results

### Infiltration rate of OS750 into enamel carious lesions

**Figure 8:**
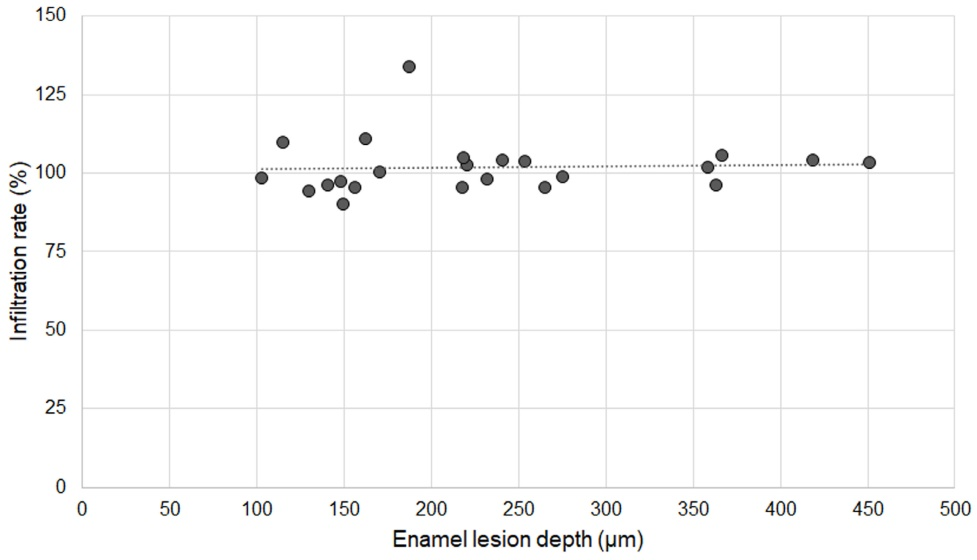
Association between infiltration rate of OS750 and enamel lesion depth.

Twenty specimens with various existing enamel lesions were used for the analysis. The average depth of enamel carious lesions tested was 232.24 µm ± 99.46 µm (102.97-451.30 µm). The infiltration rate of OS750 was 101.83% ± 8.66 (Min: 90.10%, Max: 133.94%). Spearman's correlation coefficient was 0.234 and it was not significant (p <0.05, Figure 8).

### Diagnostic accuracy of interproximal caries using NIR imaging with OS750 in an *in vitro* setting

**Figure 9:**
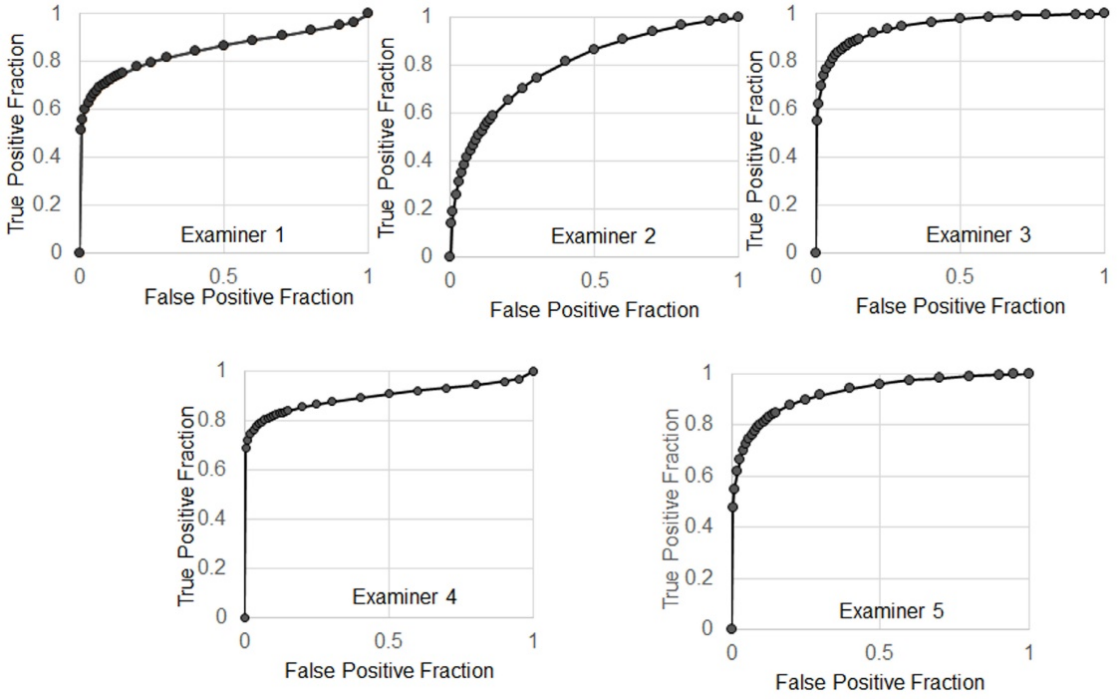
The receiver operating characteristic (ROC) curves.

The average of sensitivity and specificity of 5 examiners were 86.7% ± 4.4% and 70% ± 11%, respectively. Individual receiver operating characteristic (ROC) curves were shown in Figure 9. The average of area under the ROC curves (AUC) was 0.883 ± 0.059 indicating excellent performance.

### Diagnostic statistics of accuracy of interproximal caries by NIR imaging using OS750 in an *ex vivo* setting

The mean sensitivity of 8 examiners was 82.97% ± 15% (60.87~100) and 76.78% ± 13.27% (60~93.75) for specificity (Table 1). Cohen's κ of that 2 dentists repeated the assessment 3 times with 1 week in between 0.737. The inter-examiner agreement among 8 examiners was shown in Table 2. The mean Cohen's κ was 0.427 ± 0.138.

### Progression of enamel carious lesions after etidronate application

**Figure 10:**
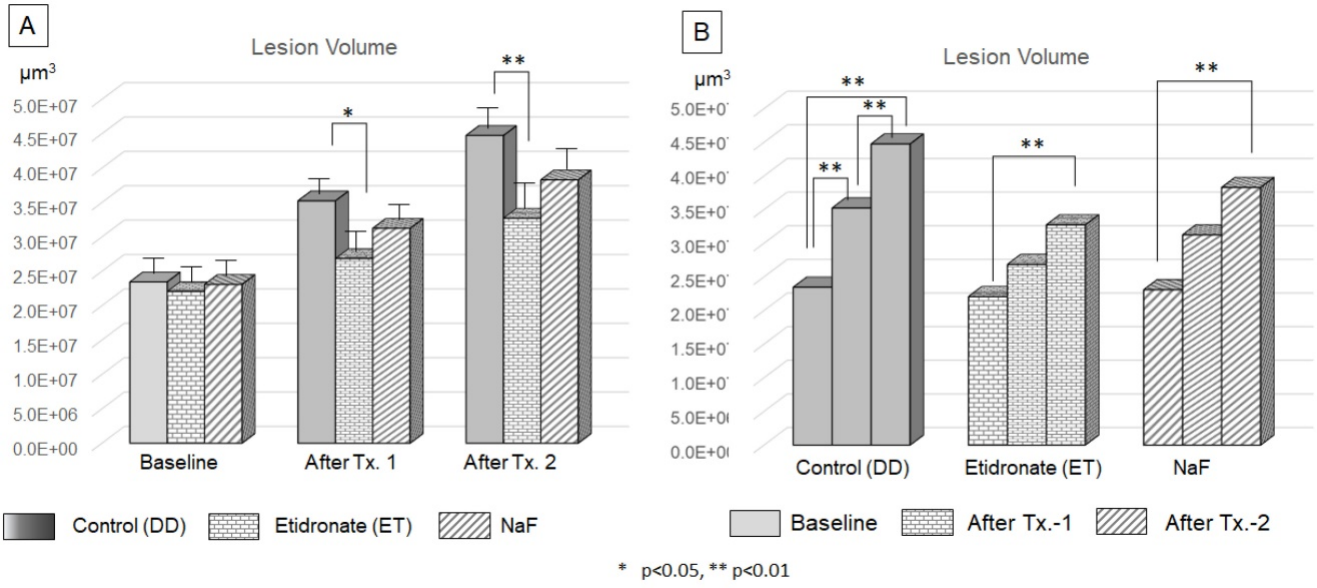
** Efficacy of Etidronate and NaF on the progression of carious lesion volume.** The two-way ANOVA and Tukey's HDS post-hoc test indicated significant difference among treatment type **(A)** and treatment period **(B)**.

The volume of lesions at the baseline, after treatment-1 and treatment-2 were shown Figure 8. ANOVA and Tukey's HDS post-hoc test indicated significant difference among time point of treatment and treatment materials. The lesion volume treated by Etidronate was significantly smaller at after treatment-1 (p<0.05) and treatment-2 (p<0.01) than the control (Figure 10A). For the control group, lesion volume increased significantly along with 3 demineralization periods (p<0.01). In contrast, for Etidronate and NaF group, lesion volume indicated no significant difference on the time point of after treatment-1 (Figure 10B).

## Discussion

Near-InfraRed-Fluorescence (NIRF) with OS750 has been widely used for diagnostic imaging of osteoblastic activity in animals, but it has never been applied in dentistry for diagnostic imaging of caries 11. Our previous study confirmed the use of NIRF as a promising method for caries imaging. OS750 was shown to selectively target dental caries with a 93% infiltration rate which was an advantage for NIRF detection and imaging of early caries. In this *in vitro* study, we focused on early-stage enamel carious lesions, for which preventive care should reduce further demineralization. Five calibrated operators demonstrated high sensitivity (86.7%) and AUC which is desired for the detection of early-stage caries. Tests with high sensitivity are helpful during the early stages of a diagnostic workup. Untreated caries progression will lead to irreversible carious lesions that will require surgical intervention. Many surgical interventions may require invasive tooth preparation and can ultimately lead to tooth loss. This *in vitro* and *ex vivo* study showed a lower specificity. A potential cause for lower specificity value may be false positives due to high reflected fluorescence from the lesion. The mean values of Cohen's κ was 0.427, indicating moderate inter-examiner agreement. Adjusting the level of fluorescence of OS750 could potentially increase the specificity and increase inter-examiner agreement. Disagreement among examiners could also be due to the nature of handling the face mounted device that are not very well implemented for dentistry yet. For the purpose of early caries detection, a diagnostic test with high sensitivity is more critical than specificity. Starting conservative caries prevention therapy early is beneficial and the extent of caries can be further worked up by clinical and radiographic examination and monitored over time.

Fluoride has been the standard therapy for caries prevention and control for decades. Fluoride in higher concentrations, such as fluoride varnish (5% sodium fluouride), is available and its efficacy has been studied 26-28. It has been reported that fluoride varnish is effective in protecting tooth surfaces that were sound at baseline 26. However, it failed to reduce caries development in toddlers from high-risk communities 27. Fluoride varnish had no impact on caries progression as a biannual treatment, but did protect enamel surfaces from the surface roughness of acid erosion by soft drinks. 5% fluoride varnish is only capable of remineralizing early enamel caries. The efficacy of fluoride is limited to incipient carious lesions due to its inability to penetrate deeply into enamel 29. Once the lesion progresses beyond the enamel-dentin junction, surgical dental procedures are required to prevent further tooth destruction. Despite improvements in restorative materials over the years, operative procedures result in loss of natural tooth structure, postoperative tooth sensitivity, and secondary decay may occur.

Potential side effects of fluoride have been pointed out in recent literature. Gao et al reported combined toxic effect of sodium fluoride (NaF) and sulfur dioxide (SO_2_) on kidney morphological changes and DNA damage in male Wistar rats 28. Jiang et al indicated that long-term fluoride exposure dose-dependently suppressed GSK-3b/b-catenin signaling (GSK: glycogen synthase kinase), highlighting the involvement of GSK-3b/b-catenin pathway and the downstream genes, cyclin D1 and c-myc, in the multifold of fluoride-induced neurotoxicity 30. A compound that can prevent further demineralization of deep enamel caries can provide a huge benefit to our patients by preventing or delaying surgical dental procedures.

Studies have shown that bisphosphonates are able to bind with high affinity to exposed hydroxyapatite in deep carious lesions in enamel ^16-18^. Our results indicate that bisphosphonates have a greater protective effect against demineralization. Bisphosphonates have a P-C-P chemical moiety, which gives its resistance to hydrolysis by phosphatases and a high binding affinity to the calcium atoms of hydroxyapatite crystals 17, 31. This structure is chemically similar to the P-O-P group in their natural analog pyrophosphates. *Gandolfi et al.* hypothesized that bisphosphonate may react with the enamel and form a protective layer as a diffusion barrier for exchange of calcium and phosphate ions ^16^. Due to the high affinity of bisphosphonates to calcium in hydroxyapatite, the reaction between the two is likely to be instantaneous and rapid. Burton & Neuman showed that bisphosphonates can bind to hydroxyapatite only if the crystals are exposed and accessible 2, 32. Our study showed that bisphosphates are more protective when carious lesions get deeper and more progressed. This may be due to the fact that early carious lesions have an intact surface zone with subsurface demineralization before they progress to the point that the intact zone becomes disrupted. The formation of the surface zone is not fully understood. The intact surface zone may be due to a dynamic equilibrium of the enamel surface, oral fluid, salivary proline-rich proteins, other salivary inhibitors, as well as the ultrastructural and chemical composition of the enamel surface. The intact surface zone can prevent bisphosphonates from penetrating deeply into the enamel to inhibit further demineralization. Once the caries progresses and the surface zone becomes compromised, bisphosphonates may be better able to bind with exposed hydroxyapatite and inhibit further demineralization. It is also possible that the bisphosphonate protective layer may interfere with remineralization. Two *in vitro* studies reported that bisphosphonates Etidronate HEBP and clodronate Cl_2_MBP adsorb on growing crystallites and blocked hydroxyapatite crystal growth, therefore inhibiting enamel and dentin mineralization 17, 31. HEBP and Cl_2_MBP bound to hydroxyapatite and inhibited amorphous calcium phosphate from transforming into hydroxyapatite or other calcium phosphate crystals, therefore blocking both growth and dissolution of such crystals. If the role of bisphosphonates is limited to inhibiting demineralization, perhaps they may be most efficacious when used in combination with compounds that facilitate remineralization. This would create a two-step approach to caries treatment. First step, caries arrest with bisphosphonates and the second, remineralization occurs with an agent such as fluoride.

There are important differences when bisphosphonates are used for topical application versus systemic therapy. Some known side effects of systemic bisphosphonate use are increased risks of pathologic fractures, osteonecrosis of the jaw (ONJ) and atrial fibrillation 8. Topical application has negligible amount of bisphosphonates compared to doses used in systemic bisphosphonate therapy. For the purpose of dental diagnostic imaging, OS750 is applied only topically with oral rinsing for a short period of time, compared to long-term use of systemic bisphosphonate therapy which usually has a higher dose frequency. In addition, the proposed target tissue for caries diagnosis imaging is the teeth, not bone. The systemic exposure is expected to be minimal and well below the known toxic levels. Several bisphosphonate drugs, taken by mouth, are available to treat osteoporosis. Two FDA approved Alendronate formulations (one 70-mg tablet per week and one 35-mg tablet per week) are available for osteoporosis prevention. An adult using the preventive formulation would receive 1680 mg alendronate over the course of one year. In our caries detection, 5 µl of OS750 at 1:100 dilution was applied to each tooth, which amounted to 200 pmol for 20 teeth. OS750 is a conjugate of alendronate and fluorescent dye of Cy-5. Based on the total molecular weight of 1101 of OS750 and that of 250 of alendronate, 200 pmol of OS750 corresponds to 45 pg of alendronate that is 9 digits lower than the single dose of oral alendronate.

In this study, there are limitations to consider. Improving the user-friendliness of face mount technique such as better handling and more training tools would help with the application of diagnostic tests. We artificially created white spot lesions in an environment which was limited to *in vitro* demineralization cycles. The oral cavity is a complex ecosystem with a dynamic demineralization and remineralization cycle. For the future study, broader implications including a variety of treatment sequences with different lesion volumes with deeper caries is necessary to determine the optimal number of treatments necessary to inhibit lesion progression with different degrees of demineralization.

## Conclusions

This study suggests that bisphosphonates contribute to both early diagnosis of enamel caries and inhibition of caries progression. OS750 detects early enamel caries. Etidronate preferentially binds to hydroxyapatite and penetrate deeply into enamel, promoting long-term inhibition of enamel caries progression. Bisphosphonates are a promising class of compounds that can help detect caries and inhibit progression and used as an alternative to conventional fluoride treatment.

## Figures and Tables

**Table 1:** Sensitivity and specificity of 8 examiners

Examiners	I	II	III	IV	V	VI	VII	VIII	Mean	SD
**Sensitivity**	93.75	93.75	66.67	68.75	90.00	90.00	60.87	100.0	82.97	15.01
***95% Confidence Interval***										
Lower limit	67.71	67.71	38.69	87.01	54.12	54.12	38.78	65.55		
Upper limit	99.67	99.67	87.01	87.87	99.48	99.48	79.53	100.0		
**Specificity**	93.75	93.75	60.00	68.75	68.18	68.18	88.89	72.73	76.78	13.27
***95% Confidence Interval***										
Lower limit	67.71	67.71	99.67	41.48	45.12	45.12	63.93	49.56		
Upper limit	99.67	99.67	82.54	87.87	85.27	85.27	98.05	88.39		

**Table 2:** The inter-examiner agreement among 8 examiners by Cohen's κ

Examiner	I	II	III	IV	V	VI	VII	VIII	Mean	SD
Individual Mean	0.55	0.55	0.22	0.24	0.43	0.37	0.54	0.52	0.427	0.138
SD	0.27	0.17	0.12	0.17	0.19	0.19	0.25	0.16		

SD: Standard deviation.

## Abbreviations

AUC: Area under the curve
CCD: Charge-Coupled Device
ddH2O: Doubled distilled water
EHDP: Ethane-1-hydroxyl-1, 1-diphosphonate
ET: Etidronate
GSK: Glycogen synthase kinase
ICG: Indocyanine green
MHDP: Mmethane-hydroxy-diphosphonate
NaF: Sodium fluoride
NIRF: Near-InfraRed-Fluorescence
ONJ: Osteonecrosis of the jaw
OS750: OsteoSense 750^®^
ROC: Receiver operating curves
ROI: Region of interest
